# Outcomes of Lurbinectedin in Central Nervous System Metastases of Small Cell Lung Cancer: A Single-Institution Retrospective Case Series

**DOI:** 10.3390/reports9020179

**Published:** 2026-06-07

**Authors:** Navanita Biswas, Carolin Schmidt, Shoja Rahimian

**Affiliations:** 1 Department of Internal Medicine, Tower Health Reading Hospital, West Reading, PA 19608, USA; 2 Drexel University College of Medicine, Drexel University, Philadelphia, PA 19129, USA; cs3888@drexel.edu; 3 Department of Hematology & Oncology, Tower Health Reading Hospital, West Reading, PA 19608, USA

**Keywords:** small cell lung cancer, central nervous system metastases, lurbinectedin, intracranial progression-free survival, retrospective case series, second-line therapy

## Abstract

**Background/Objectives**: Central nervous system (CNS) metastases are a frequent and morbid complication of small cell lung cancer (SCLC), with limited effective systemic treatment options. Lurbinectedin has demonstrated systemic activity in relapsed SCLC; however, its intracranial efficacy remains unclear because patients with active CNS disease were underrepresented in pivotal trials. We evaluated real-world intracranial outcomes of lurbinectedin in patients with SCLC and CNS metastases. **Methods**: A single-institution retrospective case series was conducted among adult patients with histologically confirmed SCLC and radiologic CNS metastases treated with lurbinectedin between July 2020 and April 2025. Primary endpoints were CNS disease control rate (CNS-DCR), defined as radiographic stability or improvement lasting ≥8 weeks, and intracranial progression-free survival (iPFS), defined as time from lurbinectedin initiation to clinical or radiographic CNS progression or death. **Results**: Thirty patients received lurbinectedin; 14 (46.7%) had CNS metastases at any time. Five patients (16.7%) had baseline CNS metastases prior to lurbinectedin initiation, while nine (30.0%) developed CNS metastases during treatment. Among patients with baseline CNS disease, one patient demonstrated radiographic intracranial improvement at approximately 4 months; however, systemic progression at 5 months limited further assessment of response duration. The remaining four patients experienced intracranial progression within 2–4 months. One of five patients with baseline CNS metastases met the predefined CNS disease control endpoint; this descriptive proportion corresponds to 20% within our small sample. Median iPFS was approximately 2.5 months. No CNS-specific adverse events attributable to lurbinectedin were observed. **Conclusions**: In this single-institution retrospective case series, limited intracranial disease control was observed among SCLC patients with baseline CNS metastases treated with lurbinectedin. Given the small number of evaluable patients, these findings should be interpreted as descriptive and hypothesis-generating rather than a conclusive efficacy analysis. Prospective studies incorporating CNS-specific endpoints are needed to better define the role of lurbinectedin and other systemic therapies in intracranial disease management.

## 1. Introduction

Small cell lung cancer (SCLC) is an aggressive neuroendocrine tumor that accounts for approximately 13% of all lung cancer diagnoses in the United States [1]. It is characterized by early and extensive metastasis; in a SEER-based study, Wells et al. evaluated 111,263 patients diagnosed with SCLC between 2000 and 2019 and reported that >70% had distant metastases or extensive-stage SCLC (ES-SCLC) at diagnosis [2]. Other studies have reported similar findings, indicating that approximately 60–85% of patients have ES-SCLC at diagnosis [3,4,5,6,7]. The central nervous system (CNS) is a common site of metastasis, with ~10–19% of patients having CNS metastases at diagnosis and ~40–78% developing CNS involvement later [4,5,6,7,8,9,10]. The prognosis of ES-SCLC remains poor, with a median overall survival (OS) of approximately 12–13 months with current first-line therapies (platinum–etoposide plus immunotherapy) [11,12]. Notably, these pivotal trials largely excluded patients with symptomatic or untreated CNS metastases.

Given the high risk of CNS metastasis, prophylactic cranial irradiation (PCI) is recommended for patients with limited-stage disease and for select patients with extensive-stage disease who have a good response to systemic treatment and no evidence of CNS metastasis [7]. For patients with detectable CNS metastases, whole-brain radiation therapy (WBRT) has historically been a standard approach; however, stereotactic radiosurgery (SRS) is also increasingly utilized with non-inferiority and fewer neurocognitive adverse effects demonstrated in selected patients with lower burden CNS disease on imaging [7,13,14]. Despite CNS-directed therapies, many patients relapse and require second-line systemic agents such as lurbinectedin, topotecan/irinotecan, platinum rechallenge in selected cases, or tarlatamab [6,15].

Lurbinectedin is an alkylating derivative of the marine drug trabectedin with increased clinical practice since 2020 for the treatment of patients with ES-SCLC who relapse after platinum-based chemotherapy [16]. The mechanism of action involves targeting guanine and cytosine-rich sequences in promoter regions of DNA to induce double-strand breaks as well as inhibiting RNA polymerase binding and transcription, leading to apoptosis [17]. In a phase 2 basket trial, Trigo et al. treated 105 patients with relapsed SCLC with lurbinectedin and reported an objective response rate of 35.2% and a median OS of 9.3 months [18]. However, patients with active CNS metastases were excluded [18]. Another study by Aix et al. (ATLANTIS) compared lurbinectedin plus doxorubicin to the physician’s choice of chemotherapy and found no statistically significant difference in OS or response [19]. Although patients with asymptomatic CNS metastases were allowed, intracranial outcomes were not specifically reported.

Due to the exclusion or underrepresentation of patients with CNS metastases in prospective trials, data on lurbinectedin’s intracranial activity remain limited. In this study, we aimed to evaluate real-world intracranial disease control and clinical outcomes among patients with SCLC and CNS metastases treated with lurbinectedin at a single academic institution over a five-year period. Patients with baseline CNS metastases at lurbinectedin initiation were analyzed for intracranial response outcomes, while patients who developed CNS metastases during treatment were included descriptively to characterize CNS events occurring during lurbinectedin exposure. Given the small number of patients with baseline CNS metastases, this study was designed as a descriptive, hypothesis-generating retrospective case series rather than a definitive efficacy analysis.

## 2. Methods

A single-institution retrospective case series was conducted among adult patients with histologically confirmed SCLC who received lurbinectedin at our institution between July 2020 and April 2025. Patients were included in the intracranial outcomes analysis if they had radiologic evidence of CNS metastases prior to initiation of lurbinectedin. Patients who developed CNS metastases after lurbinectedin initiation were not included in the primary intracranial response analysis because they did not have measurable baseline CNS disease at the start of treatment; these patients were retained only for descriptive characterization of CNS events during lurbinectedin exposure. Electronic medical records were reviewed to collect demographic data, Eastern Cooperative Oncology Group (ECOG) performance status, prior systemic therapies, CNS imaging findings, neurological symptoms, and survival outcomes.

All available brain MRI or CT scans performed before and during lurbinectedin treatment were evaluated. CNS disease status was determined based on radiology report assessment, with progression determined by RECIST (Response Evaluation Criteria in Solid Tumors) [20]. Details of prior brain-directed radiotherapy, including whole-brain radiation therapy (WBRT), stereotactic radiosurgery (SRS), or other focal radiation modalities, as well as the timing of radiotherapy relative to lurbinectedin initiation and subsequent CNS imaging or progression, were extracted from the electronic medical record when available.

Primary endpoints were defined by:

- CNS disease control rate (CNS-DCR): the proportion of patients who achieved radiologic stability or improvement of CNS lesions lasting ≥8 weeks after initiation of lurbinectedin.
- Intracranial progression-free survival (iPFS): time from the first dose of lurbinectedin to clinically or radiographically confirmed CNS progression or death, whichever occurred first.

Secondary data collected included extracranial progression, systemic response assessments (when available), and treatment-related adverse events, with special attention to CNS-specific toxicities.

### Statistical Analysis

Outcomes were summarized descriptively given the small sample size; iPFS was calculated from lurbinectedin initiation to intracranial progression or death, with censoring at last CNS imaging follow-up when applicable. No inferential statistics, comparative hypothesis testing, or multivariable modeling were performed due to the descriptive case-series nature of the cohort.

## 3. Results

Between July 2020 and April 2025, 30 patients with SCLC received lurbinectedin at our institution. Fourteen patients (46.7%) had evidence of CNS metastases at any point during their disease course. Five patients (16.7%) had CNS metastases at baseline (prior to lurbinectedin initiation) and comprised the analytic cohort for intracranial outcomes, while nine patients (30.0%) developed CNS involvement during treatment. Because these nine patients did not have baseline CNS metastases at lurbinectedin initiation, they were not evaluable for CNS-DCR or baseline intracranial response endpoints and were included only as a descriptive subgroup.

Baseline demographic and clinical characteristics of the five patients with baseline CNS metastases are summarized in Table 1, including age, sex, ECOG performance status, stage at diagnosis, prior systemic therapies, and prior brain-directed radiotherapy modality when available. The timing of brain-directed radiotherapy relative to lurbinectedin initiation and subsequent CNS imaging outcomes is summarized in Table 2.

**Figure 1:**
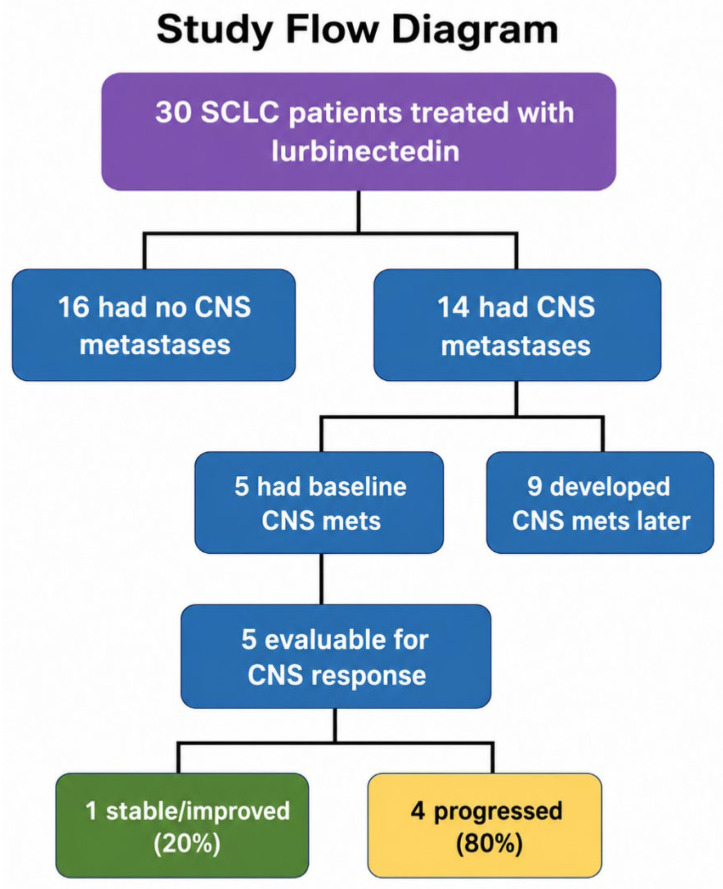
**Study flow diagram and intracranial outcomes in patients treated with lurbinectedin.** Of 30 patients with SCLC treated with lurbinectedin, 16 had no CNS metastases, and 14 had CNS metastases at any time. Among patients with CNS metastases, 5 had baseline CNS metastases at lurbinectedin initiation and were evaluable for intracranial response; 1 (20%) had stable/improved disease, and 4 (80%) progressed. Nine patients developed CNS metastases during lurbinectedin treatment and were included descriptively.

Among the five patients with baseline CNS disease, one patient (20%) demonstrated radiographic improvement in intracranial lesions at approximately 4 months following lurbinectedin initiation. However, systemic disease progression was identified at 5 months, leading to treatment discontinuation; therefore, the duration of CNS response beyond that time point could not be determined. The remaining four patients (80%) experienced radiographic CNS progression within 2–4 months of treatment initiation. One of five patients with baseline CNS metastases met the predefined CNS disease control endpoint. Given the small sample size, this finding is reported descriptively rather than as a precise estimate of treatment effect. The median intracranial progression-free survival (iPFS) was approximately 2.5 months. No CNS-specific adverse events attributable to lurbinectedin were observed. The overall cohort distribution and intracranial response outcomes are shown in Figure 1.

## 4. Discussion

CNS involvement remains a major therapeutic challenge in SCLC, with limited effective systemic options and historically poor outcomes. In this single-institution retrospective case series, we described intracranial outcomes among patients with SCLC and baseline CNS metastases treated with lurbinectedin. One of five patients met the predefined CNS disease control endpoint, and median intracranial progression-free survival was approximately 2.5 months. Given the small sample size, these findings should be interpreted descriptively and should not be considered a precise estimate of lurbinectedin’s intracranial efficacy. The nine patients who developed CNS metastases during lurbinectedin treatment were included to describe CNS events occurring during systemic therapy exposure, but they were not part of the primary intracranial response analysis. Because these patients lacked baseline measurable CNS disease, they could not be used to estimate CNS-DCR or intracranial response. Their development of CNS metastases during treatment should therefore be interpreted cautiously and does not establish whether lurbinectedin failed to prevent CNS progression. Rather, this subgroup highlights the ongoing risk of CNS involvement in SCLC and the need for prospective studies that evaluate both treatment of established CNS disease and prevention of new CNS metastases during systemic therapy.

The majority of patients with baseline CNS metastases experienced rapid intracranial progression within 2–4 months of therapy. Only one patient exhibited radiographic improvement at four months, although subsequent systemic disease progression limited the duration of clinical benefit. These findings suggest that while lurbinectedin has demonstrated systemic activity in relapsed SCLC, its effectiveness in controlling CNS disease appears limited in patients with baseline intracranial metastases.

Lurbinectedin exerts its antitumor activity primarily by binding guanine-rich sequences in the minor groove of DNA, disrupting transcriptional regulation and DNA repair pathways, and ultimately promoting tumor cell death. However, activity within CNS metastases may be limited by the unique brain metastatic microenvironment and uncertain intracranial drug exposure. Lurbinectedin is a relatively large molecule, with a molecular weight of approximately 784.87 g/mol, and reliable blood–brain barrier penetration has not been established. Although the blood–brain barrier may be disrupted in established brain metastases, blood–tumor barrier permeability is variable and may not ensure adequate therapeutic concentrations. These factors may help explain why systemic activity does not necessarily translate into durable intracranial disease control [21,22].

The available prospective evidence for lurbinectedin in SCLC provides limited guidance for patients with active CNS disease. In the phase 2 basket trial that supported lurbinectedin’s approval, patients with active CNS metastases were excluded, and intracranial-specific outcomes were not reported [18]. Similarly, although the ATLANTIS trial allowed selected patients with asymptomatic CNS metastases, the study did not provide dedicated CNS response or intracranial progression data [19]. Therefore, even small real-world case series may provide useful clinical context for populations that remain underrepresented in prospective trials.

The limited intracranial disease control observed in this small cohort may have important clinical implications. Although lurbinectedin remains an approved systemic treatment option for relapsed SCLC, our findings suggest that its intracranial activity may be limited in patients with baseline CNS metastases. Therefore, in patients with CNS-predominant relapse, lurbinectedin should not be assumed to provide reliable intracranial control, and treatment decisions should continue to incorporate brain-directed therapy, clinical status, prior radiation history, and consideration of systemic agents with stronger CNS-specific evidence when available.

Our findings underscore the need for therapies with reliable CNS activity and support further studies focused on improving intracranial disease control in relapsed SCLC. While the topoisomerase inhibitor irinotecan has demonstrated intracranial responses, the recently approved DLL3-targeted bispecific T-cell engager, tarlatamab, may also represent an important option in the evolving treatment landscape [15,23]. While, like lurbinectedin, the preliminary study for tarlatamab excluded patients with untreated CNS metastasis, emerging data suggest promising results in such patients [24,25]. Nevertheless, future studies with CNS-specific endpoints such as intracranial response rates and time to CNS progression will be helpful in supporting the use of tarlatamab and other therapies for intracranial disease in SCLC.

## 5. Limitations

Several factors may have influenced these outcomes, including the small sample size, variable CNS imaging intervals, heterogeneous prior brain-directed therapies, and the retrospective design, which may introduce selection and documentation bias. Because the baseline CNS analytic cohort included only five patients, no statistical inference regarding lurbinectedin’s intracranial efficacy can be made, and these findings should be interpreted as descriptive and hypothesis-generating rather than definitive evidence of treatment effect. Several patients also received brain-directed radiotherapy shortly before or during lurbinectedin treatment; therefore, early radiographic changes could not always be definitively distinguished from true progression, incomplete local control, radiation-related change, or pseudoprogression. This limitation is particularly relevant for patients whose CNS imaging occurred within a few months of brain-directed radiotherapy, as summarized in Table 2. Because imaging was obtained as part of routine clinical care rather than at protocol-defined intervals, progression was based on available scans and clinical documentation without systematic confirmatory imaging. Intracranial response was assessed from radiology reports rather than a prospective RANO-BM evaluation, which is specifically designed for brain metastases assessment [26]. Despite these limitations, this series provides descriptive real-world clinical context regarding lurbinectedin use in patients with SCLC and CNS metastases, a population often excluded or underrepresented in prospective clinical trials.

## 6. Conclusions

In this single-institution retrospective case series, limited intracranial disease control was observed among patients with SCLC and baseline CNS metastases treated with lurbinectedin. Although lurbinectedin remains an approved systemic treatment option for relapsed SCLC, these findings suggest that its role in CNS-predominant relapse may be limited. Given the small sample size and retrospective design, these results should be interpreted as descriptive and hypothesis-generating rather than definitive evidence of intracranial efficacy. Future prospective studies incorporating CNS-specific endpoints are needed to better define the role of lurbinectedin and other systemic therapies in patients with intracranial SCLC involvement.

## Figures and Tables

**Table 1:** **Baseline characteristics and prior therapies of patients with CNS metastases at lurbinectedin initiation (*n* = 5).**

Patient	Age (Years)	Sex	ECOG Performance Status	Stage at Diagnosis	Prior Systemic Therapies (in Sequence)	Prior Cranial Radiation/Modality
**Patient 1**	61	Female	0–1	Extensive-stage SCLC	Carboplatin/etoposide × 6 cycles; palliative thoracic radiation therapy; whole-brain radiation therapy	Yes (WBRT)
Patient 2*	**82**	**Male**	**2**	**Extensive-stage SCLC**	**Carboplatin/etoposide × 4 cycles (palliative intent)**	**Yes (WBRT)**
**Patient 3**	65	Female	0	Limited-stage SCLC (IIIA)	Cisplatin/etoposide with concurrent thoracic radiation; irinotecan/carboplatin × 5 cycles; nivolumab maintenance; carboplatin/etoposide/atezolizumab; gemcitabine; pembrolizumab	No
**Patient 4**	60	Female	0–1	Extensive-stage SCLC	Carboplatin/etoposide (cycle 1 inpatient); carboplatin/etoposide plus atezolizumab (from cycle 2); immunotherapy until progression	Yes (RT to brain metastases, modality not specified in available records)
**Patient 5**	57	Male	0–1	Extensive-stage SCLC	Carboplatin/etoposide (inpatient); carboplatin/etoposide/atezolizumab × 5 cycles; atezolizumab maintenance; lurbinectedin (second line)	Yes (SRS)

Patient 2* achieved radiographic intracranial improvement approximately 4 months after lurbinectedin initiation; systemic progression occurred at 5 months, limiting further assessment of CNS response duration.

**Table 2:** **Timing of brain-directed radiotherapy, lurbinectedin initiation, and CNS outcomes.**

Patient	Brain-Directed RT Modality	Timing of Brain RT	Lurbinectedin Initiation	CNS Imaging/Outcome Timing
**Patient 1**	WBRT	February 2023	March 2023	Progression on repeat brain MRI approximately 3 months after lurbinectedin initiation
**Patient 2**	WBRT	June 2022	July 2022	Interval resolution on repeat brain MRI approximately 4 months after lurbinectedin initiation
**Patient 4**	Brain-directed RT, modality not specified in available records	January 2025	January 2025	Disease progression in the brain in March 2025
**Patient 5**	SRS	SRS in June 2021	August 2021	Intracranial progression in December 2021, followed by salvage WBRT

This table summarizes the timing of brain-directed radiotherapy relative to lurbinectedin initiation and subsequent CNS imaging outcomes among patients with available radiation-timing data. Patient 3 was not included because the available CNS/radiation timeline was not directly comparable.

## Data Availability

The data presented in this study are available on request from the corresponding author due to privacy concerns.
